# On The Possible Leakage of ET-RR1 Liquid Waste Tank: Hydrological and Migration Modes Studies

**DOI:** 10.1100/tsw.2005.34

**Published:** 2005-03-20

**Authors:** N. S. Mahmoud, S. T. EL-Hemamy

**Affiliations:** National Center for Safety and Radiation control, Atomic Energy Authority, Nasr City (7551), Cairo, Egypt

**Keywords:** Inshas, geophysical technique, aging settling tank, instantaneous mode

## Abstract

The first Egyptian (ET-RR1) research reactor has been in operation since 1961 at the Egyptian Atomic Energy Authority (EAEA) Inshas site. Therefore, at present, it faces a serious problem due to aging equipment, especially those directly in contact with the environment such as the underground settling tanks of nuclear and radioactive waste. The possible leakage of radionuclides from these aging tanks and their migration to the aquifer was studied using instantaneous release.This study was done based on the geological and hydrological characteristics of the site, which were obtained from the hydrogeological data of 25 wells previously drilled at the site of the reactor[1]. These data were used to calculate the trend of water levels, hydraulic gradient, and formulation of water table maps from 1993–2002. This information was utilized to determine water velocity in the unsaturated zone.Radionuclides released from the settling tank to the aquifer were screened according to the radionuclides that have high migration ability and high activity. The amount of fission and activation products of the burned fuels that contaminated the water content of the reactor pool were considered as 10% of the original spent fuel. The radionuclides considered in this case were H-3, Sr-90, Zr-93, Tc-99, Cd-113, Cs-135, Cs-137, Sm-151, Pu-238, Pu-240, Pu-241, and Am-241.The instantaneous release was analyzed by theoretical calculations, taking into consideration the migration mechanism of the various radionuclides through the soil space between the tank bottom and the aquifer. The migration mechanism through the unsaturated zone was considered depending on soil type, thickness of the unsaturated zone, water velocity, and other factors that are specific for each radionuclide, namely retardation factor, which is the function of the specific distribution coefficient of each radionuclide. This was considered collectively as delay time. Meanwhile, the mechanism of radionuclide migration during their passage in the water body of the aquifer was the main focus of this study.The degree of water pollution in the aquifer at a point of contact with the main water body of Ismailia Canal 1000 m from the reactor site was assessed for the instantaneous release by comparing the results obtained with the regulations of the standard limit of radionuclides in drinking water[2,3].

## INTRODUCTION

At the Inshas site, the first Egyptian research reactor (ET-RR1), 2.2-MW thermal capacity, went critical for the first time in the fall of 1961[4]. Its aluminum fuel rods were assembled in square arrays forming the EK-10 fuel assemblies and the cladding material was made of 1.5-mm thick aluminum alloy with 500 ppm Si, 20 ppm Mg, and 20 ppm Cu. This cladding material is not corrosion resistant[5]. The spent fuel was stored in the cooling pool (30-$m^3$ capacity) next to the reactor. The pool was filled and a new pool was constructed[5]. During the cleaning of the first pool and the transfer of 37 spent fuel assemblies to the new pool, 3 of the 37 fuel assemblies were found to have suffered severe rupture[5]. In addition, the operators expected that there were other undetectable, failed fuel assemblies since the spent fuel had been stored for periods longer than originally planned. Since the average life of these facilities is 25 years and much aluminum clad material will show signs of localized or generalized corrosion after more than 30 years, water had degraded the aluminum cladding, and the fuel assemblies were seriously corroded[6].

The water in the old pool of the reactor was highly contaminated with radionuclides that were released from the defective spent fuel assemblies. The water was pumped to an underground settling tank outside the reactor building without analysis[7]. This settling tank also received waste from the various reactor components including first circulation circuit, heat exchangers, hot cells, the reactor, and other reactor components[5]. However, this contamination of wastewater collected from the reactor components is negligible as compared to the radionuclides released from the failing spent fuel assemblies.

Most underground aged tanks are susceptible to corrosion due to several causes. Consequently, they can leak for a long duration without the operator being aware[8]. By 1996, there were 314,720 reported releases from underground storage tanks, and additionally, about 30,000 new leaks were reported each year from underground tanks used for several industrial purposes, particularly petroleum tanks[8]. Therefore, the possibility of the ET-RR1 settling tanks to corrode is high, which leads to migration of contaminated water to the underground aquifer. The contaminated water, depending on the quantity and quality of radionuclide pollution, may pose a threat to public health.

The present work is directed to assess the possible leakage of the ET-RR1 settling tank in case of corrosion damage. This situation is conservatively studied due to lack of complementary data. The radioactive liquid waste can release from the settling tank instantaneously or continuously depending on the degree of corrosion of the tank. In the present study, radionuclide releases are estimated using the instantaneous mode of release. This mode primarily gives an overall understanding of the problem. Also, it appraises the maximum pollution that can occur. On the other hand, the continuous mode of release involves several complicated issues and necessitates a separate study.

Finally, compared with the standard limit of radionuclides in drinking water[2,3], the pollution of the aquifer water was assessed at a 1000-m point from the reactor site. The 1000-m point is the nearest distance between the Inshas site and the Ismailia Canal[9]. To achieve this objective, two studies were carried out. The first study was concerned with the geological and hydrological characteristics of the reactor site. The second study was concerned with the evaluation of the type, activity, and concentration of the radionuclides reaching the 1000-m point after different time intervals. To accomplish this purpose, a systematic approach was undertaken. Initially, a screening study was performed on the radionuclide source term originally present in the settling tank. This was followed by conservative consideration of the passage time of the selected radionuclides through the unsaturated zone. Further, the migration of the radionuclides through the saturated zone (aquifer) was studied to estimate the type, concentration (Bq/l), and time of arrival of the various radionuclides at the drinking water site 1000 m from the reactor site.

The overall benefit of these considerations may provide criteria that could be used to impose certain safety procedures to prevent the possible occurrence of health hazards.

## GEOLOGICAL AND HYDROGEOLOGICAL SETTING

As a part of the Eastern Nile Delta, the Inshas area is a featureless plain bounded to the west by the Ismailia Canal and to the north by Damietta branches of the Nile as show in Fig. 1 (close to the eastern coast). It is desertic in nature. The Inshas area lies between Long. 31º20′ N and 31º40′ N and Lat. 30º10′ E and 30º25′ E.

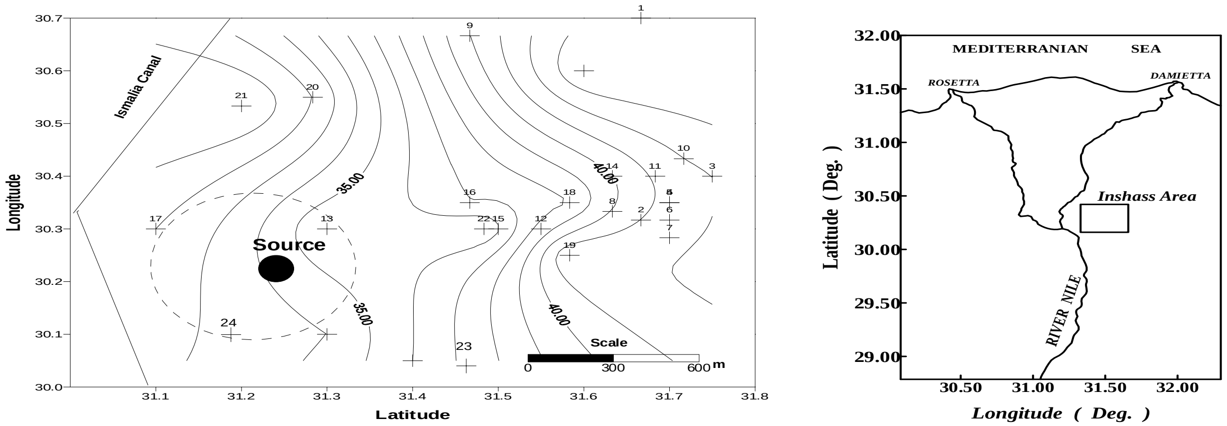

**FIGURE 1**. Location and topographic contour map of the Inshas area. (+) Test hole and piezometric well.

The surface of the area studied is covered by a thin section of Nile deposits — clays, sands, and gravels. The geology and hydrogeology of this area was introduced through the works of many authors[1,10,11,12,13,14,15,16,17].

- **Topographically,** the area investigated is not rugged, but slightly elevated over the absolute level of the water in the Ismailia Canal (about 13 m above sea level). The area has two main distinguishable features: the plateau in the east and the plain in the west.
- **Geologically**, it is covered by sedimentary rocks that belong to the Quaternary and Tertiary periods. From the correlation among the 25 test holes drilled recently in the area (Fig. 2), the Quaternary is represented by a section of Nile sediments appearing on the surface. That section is covered by a thin layer of wind-blown sands. The sediments constitute variable proportions of sand, clay, and gravel with lateral variations that are prominent and linticular in shape. The thickness of this formation is not uniform as indicated by the drill-hole loges in the study area and its vicinity. It varies between 35 m in the east, where it uncomfortably overlies the Miocene deposits, to about 10 m in the vicinity of Ismailia Canal, where it uncomfortably overlies the Mid-Tertiary basalt. On the other hand, thickness of the Quaternary deposits exceeding 62 and 68 m were reported at El-Insha and Abu-Zabaal drill holes, respectively, while in Lapor Hospital, the thickness does not exceed 19 m[13,14].
- **Structurally,** the absence of Miocene sediments in some drill holes and their exposure at the surface towards the east suggests the presence of some faults in the area. Eleven inferred fault lines crossing the area and its vicinity have been detected by the Egyptian Geological Survey. Most of these faults have the NW and NE trend. Neotectonic studies have been carried out of the area to examine the effect of these inferred faults on the Quaternary sediments covering the area of the study and its vicinity. It is clear that these faults belong to Tertiary age.

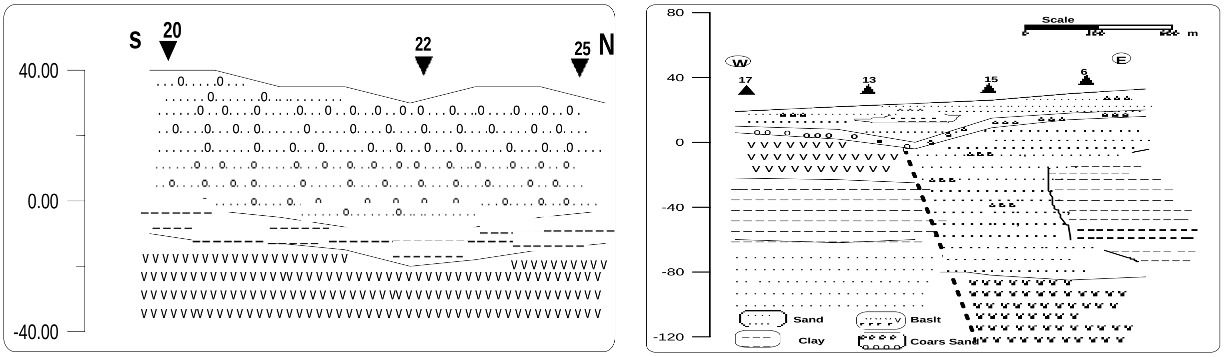

**FIGURE 2.** (Left) Geologic cross-section at NS Inshas area; (right) geologic cross-section at EW Inshas area.

- **Hydrogeologically**, from the geoelectrical and test holes of the area, the absolute level of the groundwater ranges from 12.5–13 m above sea level, which coincides with the level of the surface water at Inshas[11]. This fact indicates that the groundwater at Inshas is connected with the Ismailia Canal (Nile water).

It is clear from the cross-sections shown in Fig. 2 that the surficial aquifer is the Quaternary water-bearing formation. This aquifer occupies the western sector of the area studied west of the NW fault. It is composed of loose sand rounded to subrounded, very coarse to fine, well sorted, occasionally with gravel lenses that accelerate the percolation of groundwater to be detected in shallow wells. The vertical dimension of this aquifer ranges from 16 m in the extreme west to about 23 m. It is underlined by a thin bed of clay followed by basalt. The Quaternary aquifer at the site is bounded by the Ismailia Canal to the west and the Miocene sediments to the east. No north or south boundaries are present. Fluctuations in the level of the groundwater have been observed. These fluctuations are moderate since the groundwater is connected to the flow-controlled river system[12].

The permeability coefficients range from $4.9 \times 10^{-3}$ cm/sec to $8.1 \times 10^{-3}$ cm/sec. Generally, a value of $4.9 \times 10^{-3}$ cm/sec indicates a low permeable aquifer. The transmissibility of the aquifer ranges from 56.7–100 $m^3$/day/m at the prevailing water temperature[17].

## Field Work

In the area of study, the groundwater situation was assessed through 3 water table maps for the Quaternary aquifers using 16 wells that were drilled by the Research Institute for Water Resources in 1993; the other wells were drilled by the Egyptian Geological Survey and Mining Authority in 2000. Groundwater was collected from the wells in and around Inshas during the period 1993–2002[15]; 25 water samples were collected from those wells. Some of those data were collected by the Egyptian Geological Survey (2003, 2000). A water table map was constructed for those wells tapping the Quaternary aquifer. These maps revealed the following.

### *During 1993*

As observed from the wells, geological cross-section, and water table map, it is strongly believed that the porous Quaternary aquifers are in hydraulic connection with the Ismailia Canal, which represents the main source of the groundwater in the area. These aquifers are at a depth of about 13.5 m from the surface. Fig. 3 designs the quaternary aquifers. These maps revealed the following:

- The direction of groundwater flow is regionally towards the NW, i.e., towards the Ismailia Canal.
- The Ismailia Canal is the main source of the recharge.
- The faults crossing the site have their extension crossing Ismalia Canal outside the site. These faults are responsible for recharging of the aquifer, besides the lateral seepage from Ismailia Canal.
- The hydraulic gradient ranges from 0.0006–0.015 m/m.

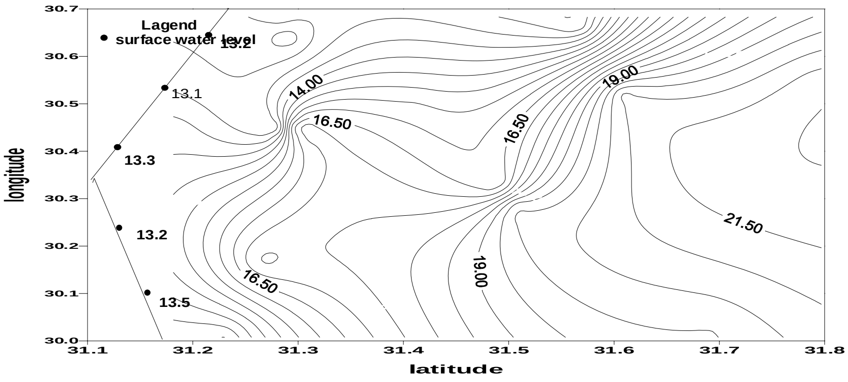

**FIGURE 3.** Water table map of the Inshas area (1993).

## *During 2000*

During this period, there was an increase of population around the site under study, which was expected to increase the groundwater extraction. The water table map for this period (Fig. 4) shows the following remarks.

- The direction of the groundwater is the same as that of 1993.
- The hydraulic gradient shows a little variation. It ranges from 0.0008–0.015 m/m.

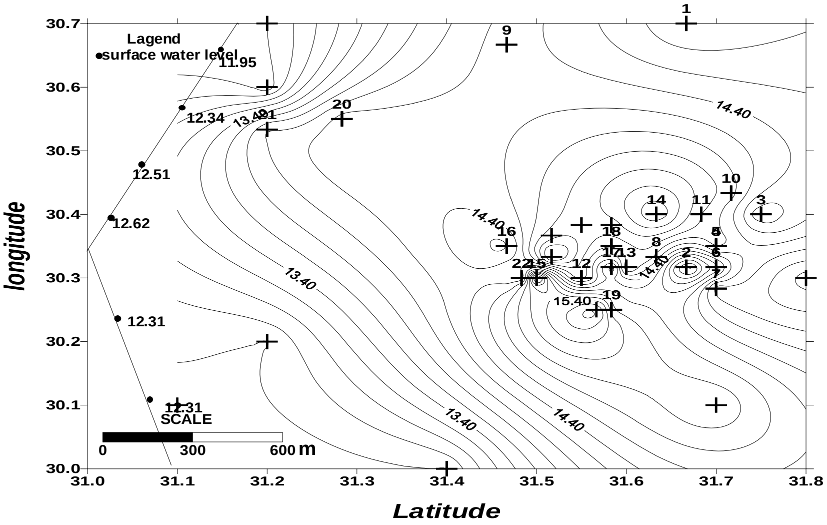

**FIGURE 4.** Water table map of the Inshas area (2000).

### *During 2002*

- The direction of the groundwater is the same as that of 2000 (see Fig. 5).
- The hydraulic gradient shows a little variation; it ranges from 0.0005–0.013 m/m.

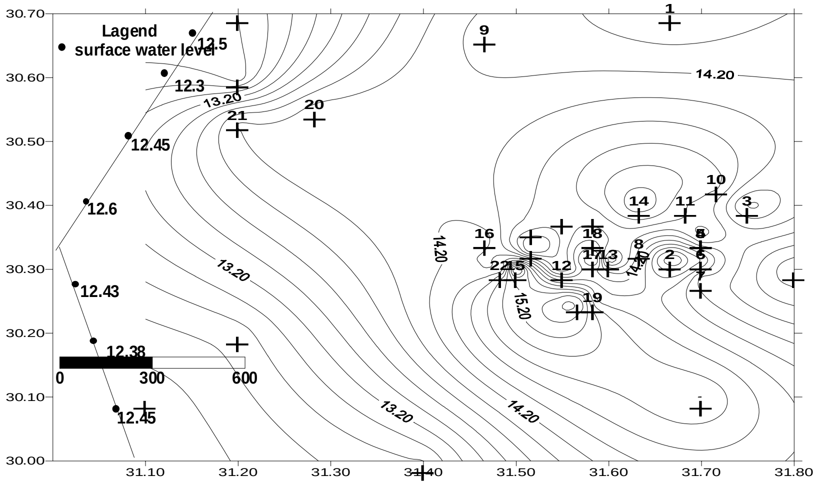

**FIGURE 5**. Water table map of the Inshas area (2002).

## SCREENING PROCESS

Several radionuclides resulted from the fission and activation processes of the fuel during reactor operation. These had variable degrees of solubility in the water of the reactor pool, which was eventually pumped to the settling tank. The ultimate aim of the study was to consider the concentration of radionuclides that would be released from the settling tank in case of corrosion damage and that would reach the drinking water site at approximately 1000 m to the west of the location of the settling tank. The concentration of these radionuclides was assessed according to the standard limit of radionuclides in drinking water[2,3].

The source term considered is the initial radioactivity calculated based on 20-year working conditions of fuel assemblies and 20-years cooling period. These conditions are reported in the Pre-Construction Safety Analysis Report[5]. During the 20-year reactor working period, 37 fuel assemblies were spent and were stored in the cooling pool for 20 years. This pool of 30-m$^3$ capacity was filled and a new pool was constructed[5]. During cleaning of the old pool and the transfer of 37 spent fuel assemblies to the new pool, 3 of the 37 fuel assemblies were found to have suffered severe rupture[5]. The water in the old pool was pumped to a settling tank originally constructed with the reactor. From the source term, only 10% of the total activity of each radionuclide was considered in the study. Table 1 shows the activity in each assembly, the total activity in 37 assemblies (source term), the 10% of the total activity, the concentration in Bq/m$^3$, and the half-life of each radionuclide.

Not all radionuclides reach the point of the concerned aquifer with high concentration. Therefore, a screening process should be done for the radionuclides present in the settling tank (10% of source term). This study focused essentially on estimating radionuclide concentrations that would reach the 1000-m site higher than the standard limit of drinking water.

**TABLE 1**
**The Source Term and Parameters of Radionuclides Considered in this Study**

| Radionuclide | Half-Life | Activity in One Assembly (Bq)[5] | Total Activity in 37 Assemblies* (Bq) | 10% from Total Activity (Bq) | Concentration (Bq/m$^3$) |
|---|---|---|---|---|---|
| Pu-238 | 8.78E + 01 year | 4.72E + 09 | 1.75E + 11 | 1.75E + 10 | 5.83E + 08 |
| Pu-239 | 2.41E + 04 year | 7.60E + 09 | 2.81E + 11 | 2.81E + 10 | 9.37E + 08 |
| Pu-240 | 6.54E + 03 year | 3.68E + 09 | 1.36E + 11 | 1.36E + 10 | 4.53E + 08 |
| Pu-241 | 1.44E + 01 year | 2.02E + 11 | 7.49E + 12 | 7.49E + 11 | 2.50E + 10 |
| Am-241 | 4.33E + 02 year | 8.45E + 09 | 3.13E + 11 | 3.13E + 10 | 1.04E + 09 |
| Am-242 | 1.52E + 02 year | 3.84E + 07 | 1.42E + 09 | 1.42E + 08 | 4.73E + 06 |
| Cm-242 | 1.63E + 02 day | 3.16E + 07 | 1.17E + 09 | 1.17E + 08 | 3.90E + 06 |
| H-3 | 1.24E + 01 year | 4.64E + 09 | 1.72E + 11 | 1.72E + 10 | 5.72E + 08 |
| Kr-85 | 1.07E + 01 year | 1.04E + 11 | 3.85E + 12 | 3.85E + 11 | 1.28E + 10 |
| Sr-90 | 2.91E + 01 year | 1.75E + 12 | 6.47E + 13 | 6.47E + 12 | 2.16E + 11 |
| Y-90 | 2.66E + 00 day | 1.75E + 12 | 6.47E + 13 | 6.47E + 12 | 2.16E + 11 |
| Zr-93 | 1.53E + 06 year | 6.03E + 07 | 2.23E + 09 | 2.23E + 08 | 7.43E + 06 |
| Tc-99 | 2.13E + 05 year | 4.02E + 08 | 1.49E + 10 | 1.49E + 09 | 4.96E + 07 |
| Ru-106 | 1.01E + 00 year | 5.53E + 07 | 2.05E + 09 | 2.05E + 08 | 6.82E + 06 |
| Rh-106 | 2.99E + 01 sec | 5.53E + 07 | 2.05E + 09 | 2.05E + 08 | 6.82E + 06 |
| Cd-113 | 1.46E + 01 year | 2.38E + 08 | 8.80E + 09 | 8.80E + 08 | 2.93E + 07 |
| Sb-125 | 2.77E + 00 year | 2.77E + 09 | 1.02E + 11 | 1.02E + 10 | 3.41E + 08 |
| Te-125 | 5.80E + 01 day | 6.75E + 08 | 2.50E + 10 | 2.50E + 09 | 8.33E + 07 |
| Cs-134 | 2.06E + 00 year | 3.88E + 09 | 1.44E + 11 | 1.44E + 10 | 4.79E + 08 |
| Cs-135 | 2.30E + 06 year | 3.46E + 07 | 1.28E + 09 | 1.28E + 08 | 4.27E + 06 |
| Cs-137 | 3.00E + 01 year | 1.91E + 12 | 7.06E + 13 | 7.06E + 12 | 2.35E + 11 |
| Ba-137 | 2.55E + 00 min | 1.80E + 12 | 6.67E + 13 | 6.67E + 12 | 2.22E + 11 |
| Pm-147 | 2.63E + 00 year | 8.09E + 10 | 2.99E + 12 | 2.99E + 11 | 9.98E + 09 |
| Sm-151 | 9.01E + 01 year | 1.99E + 10 | 7.34E + 11 | 7.34E + 10 | 2.45E + 09 |
| Eu-152 | 1.36E + 01 year | 1.16E + 09 | 4.29E + 10 | 4.29E + 09 | 1.43E + 08 |
| Eu-154 | 8.61E + 00 year | 1.28E + 10 | 4.74E + 11 | 4.74E + 10 | 1.58E + 09 |
| Eu-155 | 4.93E + 00 year | 6.34E + 09 | 2.35E + 11 | 2.35E + 10 | 7.82E + 08 |

* Source term.

## Screening Criteria

The screening criteria were based on the radionuclides that conform with any of the following considerations:

- Radionuclides in the form of gas
- Radionuclides with half-lives in seconds, minutes, or days

Six radionuclides are excluded according to those two criteria; namely Cm-242, Kr-85, Y-90, Rh-106, Te-125, and Ba-137. Radionuclides remaining after the two previous criteria undergo considerable decay during passage through both unsaturated and saturated zones and reach the 1000-m distance with concentrations below the standard limit of radionuclides in drinking water[2,3]. This particular criterion is performed through conservative mathematical model. Radionuclides considered for this screening model are presented in Table 2.

**TABLE 2**
**Radionuclides Considered in the Screening Model**

| Radionuclide | Half-Life | Concentration ($Bq/m^3$) |
|---|---|---|
| Pu-238 | 8.78E + 01 year | 5.83E + 08 |
| Pu-239 | 2.41E + 04 year | 9.37E + 08 |
| Pu-240 | 6.54E + 03 year | 4.53E + 08 |
| Pu-241 | 1.44E + 01 year | 2.50E + 10 |
| Am-241 | 4.33E + 02 year | 1.04E + 09 |
| Am-242 | 1.52E + 02 year | 4.73E + 06 |
| H-3 | 1.24E + 01 year | 5.72E + 08 |
| Sr-90 | 2.91E + 01 year | 2.16E + 11 |
| Zr-93 | 1.53E + 06 year | 7.43E + 06 |
| Tc-99 | 2.13E + 05 year | 4.96E + 07 |
| Ru-106 | 1.01E + 00 year | 6.82E + 06 |
| Cd-113 | 1.46E + 01 year | 2.93E + 07 |
| Sb-125 | 2.77E + 00 year | 3.41E + 08 |
| Cs-134 | 2.06E + 00 year | 4.79E + 08 |
| Cs-135 | 2.30E + 06 year | 4.27E + 06 |
| Cs-137 | 3.00E + 01 year | 2.35E + 11 |
| Pm-147 | 2.63E + 00 year | 9.98E + 09 |
| Sm-151 | 9.01E + 01 year | 2.45E + 09 |
| Eu-152 | 1.36E + 01 year | 1.43E + 08 |
| Eu-154 | 8.61E + 00 year | 1.58E + 09 |
| Eu-155 | 4.93E + 00 year | 7.82E + 08 |

## Mathematical Treatment for Screening Model

The screening model is based on conservative assumptions related to the passage of radionuclides in both unsaturated and saturated zones without any interaction effects (chemical reaction, dilution, advection, diffusion, etc.) until they reach a point 1000 m from the settling tank. Only the delay time due to soil sorption and decay is considered.

This delay time (travel time) results in the decrease of concentration of each radionuclide depending on its half-life. At the distance of concern, the concentration calculated for each radionuclide was compared to the standard limit of drinking water. Radionuclides with concentrations less than or equal to the standard limit were screened.

According to the hydrological and geological studies, the data of unsaturated and saturated zones required for calculations in this study are presented in Table 3.

The calculations in the screening model involve several sequential considerations, namely, seepage velocity in unsaturated and saturated zones, retardation factor in both zones, travel time in both zones, and decay process of the radionuclides during their passage in both zones to reach the point of concern at 1000 m[20].

**TABLE 3**
**The Hydrological Parameters of the Site**

| Factor | Value[17] |
|---|---|
| Vertical distance of unsaturated zone(x) | 13 m |
| Velocity of flow in the unsaturated zone (v) | 0.0039 m/sec |
| Moisture content of the sand soil (θ) | 0.25 |
| Density of the sand soil (ρ) | 2.6 $g/cm^3$ [19] |
| Seepage velocity in the aquifer | 0.04 m/day |
| Dispersion coefficient in x direction $D_x$ in the aquifer | 268.3 $m^2$/year |
| Dispersion coefficient in y direction $D_y$ | 134.1 $m^2$/year |

## *Calculation of Seepage Velocity*

$U_u$ and $U_s$ (m/year) for both zones are determined by the following:

$$U_u = v_u/\theta_u \qquad U_s = v_s/\theta_s$$

where $\nu_u$ and $\nu_s$ = velocity of radionuclide in unsaturated and saturated zone (m/year), $\theta_u$ and $\theta_s$ = average moisture content of the soil in unsaturated and saturated zone (dimensionless).

## *Calculation of Retardation Factor*

$R_{du}$ and $R_{ds}$ in unsaturated and saturated zone (dimensionless):

$$R_{du} = 1 + (\rho_u \,.\, K_{du}/\theta_u) \qquad R_{ds} = 1+ (\rho_s \,.\, K_{ds}/\theta_s)$$

where $\rho_{\mathbf{u}}$ and. $\rho_s$ = bulk density of soil in unsaturated and saturated zones ($kg/m^3$), $K_{du}$ and $K_{ds}$ = distribution coefficient of the radionuclide in unsaturated and saturated zones ($m^3$/kg).

$$K_d = \frac{\text{Amount of radionuclide adsorbed (Bq) per kilogram of soil}}{\text{Amount of radionuclide (Bq) per cubic meter of water}}$$

For safety assessment calculations, it is assumed that the adsorption of radionuclides on soil is a reversible linear relation[21]. Therefore, the distribution coefficient ($K_d$) was used as constant value for each radionuclide. Since the type of soil is similar in both unsaturated and saturated zones, the distribution coefficient for each radionuclide was considered the same in both zones.

## *Calculation of Travel Time*

$T_u$ ,$T_s$ for both unsaturated and saturated zone (y):

$$T = T_u + T_s \qquad T_u = d_{xu} \,.\, R_{du}/U_u \qquad T_s= d_{xs} \,.\, R_{ds}/U_s$$

where $d_{xu}$ and $d_{xs}$ = distance traveled by the radionuclide in unsaturated and saturated zones (m), $u_u$ and $u_s$ = seepage velocity of radionuclides in unsaturated and saturated zone (m/year).

The concentration of each radionuclide C reaching at 1000 m distance from the reactor site to the aquifer was calculated using the equation[20].

## Calculation of Decay Process

Resulted from the travel time of radionuclide through both unsaturated and saturated zone. This process decreases the concentration of radionuclide from $C_i$ to C and is calculated using the following equation:

$$C = C_i.e^{-\lambda T} \quad (1)$$

where C = concentration of radionuclide reaching 1000-m distance in the aquifer (Bq)r; $C_i$ = initial concentration of radionuclides (Bq), e = base of natural logarithm, $\lambda$ =decay constant (year$^{-1}$), and

$$\lambda = \text{Ln } 2/\, t_{1/2}$$

where $t_{1/2}$ = radionuclide half-life (year).

The results obtained from Eq. (1) and compared with the standard limits of radionuclides in drinking water are shown in Table 4.

**TABLE 4**
**Results of Screening Calculations of Radionuclides**

| Radionuclide | $K_d$[22]* ($m^3$/Kg) | $R_d$** | Travel Time in Unsaturated Zone (Year) | Travel Time in Saturated Zone (Year) | Total Travel Time (Year) | Concentration After Total Travel Time (Bq/$m^3$) | Limit Standards Drinking Water (Bq/$m^3$) |
|---|---|---|---|---|---|---|---|
| Pu-238 | 3.00E – 01 | 3.12E + 03 | 6.43E + 01 | 7.08E + 02 | 7.72E + 02 | 1.31E + 06 | 7.40E + 02 |
| Pu-239 | 3.00E – 01 | 3.12E + 03 | 6.43E + 01 | 7.08E + 02 | 7.72E + 02 | 9.17E + 08 | 7.40E + 02 |
| Pu-240 | 3.00E – 01 | 3.12E + 03 | 6.43E + 01 | 7.08E + 02 | 7.72E + 02 | 4.18E + 08 | 7.40E + 02 |
| Pu-241 | 3.00E – 01 | 3.12E + 03 | 6.43E + 01 | 7.08E + 02 | 7.72E + 02 | 1.81E – 06 | 7.40E + 02 |
| Am-241 | 9.00E – 01 | 9.36E + 03 | 1.93E + 02 | 2.12E + 03 | 2.32E + 03 | 2.56E + 07 | 7.40E + 02 |
| Am-242 | 9.00E – 01 | 9.36E + 03 | 1.93E + 02 | 2.12E + 03 | 2.32E + 03 | 1.86E – 07 | 1.85E + 06 |
| H-3 | 0.00E + 00 | 1.00E + 00 | 2.06E – 02 | 2.27E – 01 | 2.47E – 01 | 5.64E + 08 | 3.70E + 07 |
| Sr-90 | 1.50E – 02 | 1.57E + 02 | 3.24E + 00 | 3.56E + 01 | 3.88E + 01 | 8.56E + 10 | 1.85E + 04 |
| Zr-93 | 6.00E – 01 | 6.24E + 03 | 1.29E + 02 | 1.42E + 03 | 1.54E + 03 | 7.43E + 06 | 1.48E + 06 |
| Tc-99 | 1.00E – 04 | 2.46E + 00 | 5.06E – 02 | 5.57E – 01 | 6.08E – 01 | 4.96E + 07 | 2.22E + 06 |
| Ru-106 | 5.50E – 02 | 5.73E + 02 | 1.18E + 01 | 1.30E + 02 | 1.42E + 02 | 3.87E – 36 | 1.11E + 05 |
| Cd-113 | 2.38E – 02 | 2.49E + 02 | 5.12E + 00 | 5.64E + 01 | 6.15E + 01 | 1.59E + 06 | 1.48E + 04 |
| Sb-125 | 4.50E – 02 | 4.69E + 02 | 9.67E + 00 | 1.06E + 02 | 1.16E + 02 | 8.42E – 05 | 1.11E + 06 |
| Cs-134 | 1.00E – 01 | 1.04E + 03 | 2.15E + 01 | 2.36E + 02 | 2.58E + 02 | 1.12E – 29 | 3.33E + 04 |
| Cs-135 | 1.00E – 01 | 1.04E + 03 | 2.15E + 01 | 2.36E + 02 | 2.58E + 02 | 4.27E + 06 | 3.70E + 05 |
| Cs-137 | 1.00E – 01 | 1.04E + 03 | 2.15E + 01 | 2.36E + 02 | 2.58E + 02 | 6.14E + 08 | 3.70E + 04 |
| Pm-147 | 2.40E – 01 | 2.50E + 03 | 5.15E + 01 | 5.66E + 02 | 6.18E + 02 | 1.97E – 61 | 2.59E + 06 |
| Sm-151 | 2.40E – 01 | 2.50E + 03 | 5.15E + 01 | 5.66E + 02 | 6.18E + 02 | 2.12E + 07 | 7.40E + 06 |
| Eu-152 | 2.40E – 01 | 2.50E + 03 | 5.15E + 01 | 5.66E + 02 | 6.18E + 02 | 3.04E – 06 | 3.70E + 05 |
| Eu-154 | 2.40E – 01 | 2.50E + 03 | 5.15E + 01 | 5.66E + 02 | 6.18E + 02 | 4.00E – 13 | 2.59E + 05 |
| Eu-155 | 2.40E – 01 | 2.50E + 03 | 5.15E + 01 | 5.66E + 02 | 6.18E + 02 | 3.02E – 29 | 1.85E + 06 |

***** Distribution coefficient of radionuclide in soil; ** retardation factor.

Twelve radionuclides show higher concentrations than the standard limit in drinking water. These radionuclides are the subject of the migration study. These are H-3, Sr-90, Zr-93, Tc-99, Cd-113, Cs-135, Cs-137, Sm-151, Pu-238, Pu-240, Pu-241, and Am-241and are listed in Table 5.

**TABLE 5**
**Radionuclides that Resulted after Screening Process**

| Radionuclide | Half-Life (Year) | Initial Concentration of Radionuclide (Bq/m$^3$) |
|---|---|---|
| H-3 | 1.24E + 01 | 5.72E + 08 |
| Sr-90 | 2.91E + 01 | 2.16E + 11 |
| Zr-93 | 1.53E + 06 | 4.43E + 06 |
| Tc-99 | 2.13E + 05 | 4.96E + 07 |
| Cd-113 | 1.46E + 01 | 2.93E + 07 |
| Cs-135 | 2.30E + 05 | 4.27E + 06 |
| Cs-137 | 3.00E + 01 | 2.35E + 11 |
| Sm-151 | 9.01E + 01 | 2.45E + 09 |
| Pu-238 | 8.78E + 01 | 5.83E + 08 |
| Pu-239 | 2.41E + 04 | 9.37E + 08 |
| Pu-240 | 6.54E + 03 | 4.53E + 08 |
| Am-241 | 4.33E + 02 | 1.04E + 09 |

## MIGRATION STUDY

The migration study was done specifically for the 12 radionuclides that resulted from the screening process. The migration study started with the calculations of the concentration of the radionuclides concerned after passage through the unsaturated zone. This was necessary in order to have an estimate of the exact concentration of the radionuclides at the time they enter the saturated zone.

### Radionuclides Migration in Unsaturated Zone

The passage of the 12 radionuclides through the unsaturated zone is a complicated process subject to various parameters that require special study. In the present study, the passage of the 12 radionuclides was subject to the estimation of the delay time through the unsaturated zone and were considered in totality as delayed time before they were considered for the migration study in the saturated zone. The initial concentration of each radionuclide ($C_i$) released from the settling tank decreases to $C_o$ by the effect of the time that it takes to travel through the unsaturated zone. The travel time (delay time) in the unsaturated zone for the 12 radionuclides concerned was equal ($T_u$) as previously calculated in Table 4. The concentration ($C_o$) will be considered as the initial concentration entering the saturated zone. It was calculated by replacing T with $T_u$ in Eq. (1), which becomes:

$$C_o = C_i\, e^{-\lambda Tu} \qquad (2)$$

The typical values of the radionuclide concentration entering the saturated zone are presented in Table 6.

**TABLE 6**
**Radionuclides Considered for the Migration Process in Saturated Zone**

| Radionuclide | Initial Concentration ($Bq/m^3$) | Half-Life (Year) | Calculated Travel Time in Unsaturated Zone (Year) | Concentration of Radionuclide after Traveling Through Unsaturated Zone* ($Bq/m^3$) |
|---|---|---|---|---|
| H-3 | 5.72E + 08 | 1.24E + 01 | 0.02 | 5.71E + 08 |
| Sr-90 | 2.16E + 11 | 2.91E + 01 | 3.2 | 2.00E + 11 |
| Zr-93 | 4.43E + 06 | 1.53E + 06 | 128.6 | 4.43E + 06 |
| Tc-99 | 4.96E + 07 | 2.13E + 05 | 0.05 | 4.96E + 07 |
| Cd-113 | 2.93E + 07 | 1.46E + 01 | 5.1 | 2.30E + 07 |
| Cs-135 | 4.27E + 06 | 2.30E + 05 | 21.5 | 4.27E + 06 |
| Cs-137 | 2.35E + 11 | 3.00E + 01 | 21.5 | 1.43E + 11 |
| Sm-151 | 2.45E + 09 | 9.01E + 01 | 51.5 | 1.65E + 09 |
| Pu-238 | 5.83E + 08 | 8.78E + 01 | 64.3 | 3.51E + 08 |
| Pu-239 | 9.37E + 08 | 2.41E + 04 | 64.3 | 9.35E + 08 |
| Pu-240 | 4.53E + 08 | 6.54E + 03 | 64.3 | 4.50E + 08 |
| Am-241 | 1.04E + 09 | 4.33E + 02 | 192.9 | 7.64E + 08 |

* Concentration of radionuclide entering the saturated zone.

## Radionuclide Migration in Saturated Zone (Aquifer)

The concentration of each radionuclide entering the saturated zone is given in Table 6. Several features should be mentioned in order to give a comprehensive understanding about the methodology of radionuclide migration in the saturated zone. The most important of these features are the viscosity and density of the contents of the settling tank, chemical reactions, the final exact chemical form in which the radionuclides were released from the settling tank, and the chemical interaction and changes that might have occurred during the passage of the radionuclides through the unsaturated zone. It is very difficult to formulate a suitable model involving all these parameters mentioned. These parameters will affect the dilution of radionuclide concentration entering the saturated zone, which adds to the difficulty of the issue. Therefore, reaching the aquifer (saturated zone), the migration of these radionuclides has been analyzed without consideration of the dilution process and the chemical interactions. The proper examination of these parameters should be considered in a special study.

The factors that were considered in the study of the migration of the radionuclides in the saturated zone are advection, dispersion, retardation, and radioactive decay. These parameters were studied using an analytical model. This model is based on the understanding that radionuclide migration in the saturated zone will take the form of a plume. The concentration of these radionuclides in the plume is assumed to be homogeneous. The purpose of the migration study was to calculate the concentration of each radionuclide at certain distances and at certain times in the aquifer. Due to the undefined form of the plume, several differential equations should be used to calculate the migration of the plume as well the migration of radionuclides in the aquifer. The concept of the "Green Functions" was used to approximate the inhomogeneous form of the plume to a suitable homogeneous geometric form in order to solve one differential equation with define boundary conditions[23].

The plume formation for each radionuclide was considered in an X, Y, and Z direction, while the dispersion was considered in the X and Y direction. The analytical model that was used to formulate the differential equations of the flow in the saturated zone is based on the following assumptions[20]:

- The flow of radionuclides is homogeneous and is represented as a linear source of length b.
- The aquifer is isotropic homogeneous soil.
- The dispersion and distribution coefficients of the radionuclides are taken as constant values over the period of time that the radionuclides will take to reach the point at 1000 m distance.

The concentration (Cs) in Bq/m3 of each radionuclide in the aquifer at 1000 m will be given by[20]:

$$Cs = \frac{C_o}{n_e . R_{ds}} XYZ.e^{-\lambda t} \tag{3}$$

where $C_o$ = concentration of radionuclide entering the saturated zone (Bq/m$^3$), $n_e$ = effective porosity of saturated zone (dimensionless), $R_{ds}$ = retardation factor of radionuclide in the saturated zone (dimensionless), calculated by:

$$R_{ds} = 1 + (\rho_s \,.\, K_{ds}/\theta_s)$$

where $\rho_s$ = bulk density of soil in unsaturated and saturated zone (kg/m$^3$), $K_{du}$ = distribution coefficient of the radionuclide in the saturated zone (m$^3$/kg), $\theta_s$ = average moisture content of the soil in the saturated zone (dimensionless).

**X** = function of time and distance in x direction given by Eq. (a):

$$X = \frac{1}{\sqrt{4\pi D_x t / R_{ds}}} \exp\left[ -\frac{(d_x - ut/R_{ds})^2}{4 D_x t / R_{ds}} \right] \tag{a}$$

**Y** = function of time and distance in y direction given by Eq. (b):

$$Y = \frac{1}{\sqrt{4\pi D_y\, t / R_{ds}}} \exp\left[ -\frac{{d_y}^2}{4 D_y t / R_{ds}} \right] \tag{b}$$

where $D_x$ = dispersion coefficient in the x direction (m$^2$/year), $D_y$ = dispersion coefficient in the y direction (m$^2$/year), $d_x$ = distance in x direction (m), which equals $d_y$ = distance in y direction (m), t = time (years).

**Z** = factor depending on flow form:

$$Z = \frac{1}{b}$$

where b = length of vertical flow line (m).

**e** = base of natural logarithm and $\lambda$ = the decay constant (year$^{-1}$):

$$\lambda = \text{Ln } 2 / t_{1/2}$$

where $t_{1/2}$ = the radionuclide half-life (year).

## RESULTS AND DISCUSSION

From the boreholes data and geological study, the area under study consists of two zones. The first is the upper dry zone, mainly of sand and shaley sand with little silt and shaley sand, and the second is the water-bearing zone, mainly of sand, shaley sand, and basalt with shale interclinations. The Nile silt decreases from west to east. The thickness of the unsaturated zone (sand and shaley sand) increases from west to the east and overlies the water-bearing zone. The water table map study shows that a remarkable variation in the hydraulic gradient ranges from 0.001–0.015 in the eastern part toward the Ismailia Canal, while it ranges from 0.0005–0.0008 in the western part of the water table map. The Quaternary aquifer occupies the western sector of the area studied west of the NW fault. The thickness of this aquifer in the studied area ranges about 48 m. The groundwater elevation has a moderate change with time. Additionally, the presence of faults and the increase of pumping rate in the surrounding area increases groundwater velocity and, hence, the movement of radionuclides flow to longer distances.

Along the path of the aquifer, the concentration of radionuclides in the flow decreases gradually with time and distance. This decrease results from the retention of radionuclides in soil and from the two-dimensional dispersion of the flow in the unidirectional manner of groundwater in the aquifer. This dispersion, also, spread the migrated flow in a number of consecutive waves that shape the concentration distribution of radionuclides in the aquifer until the depletion of this flow[24], as is shown in Fig. 6.

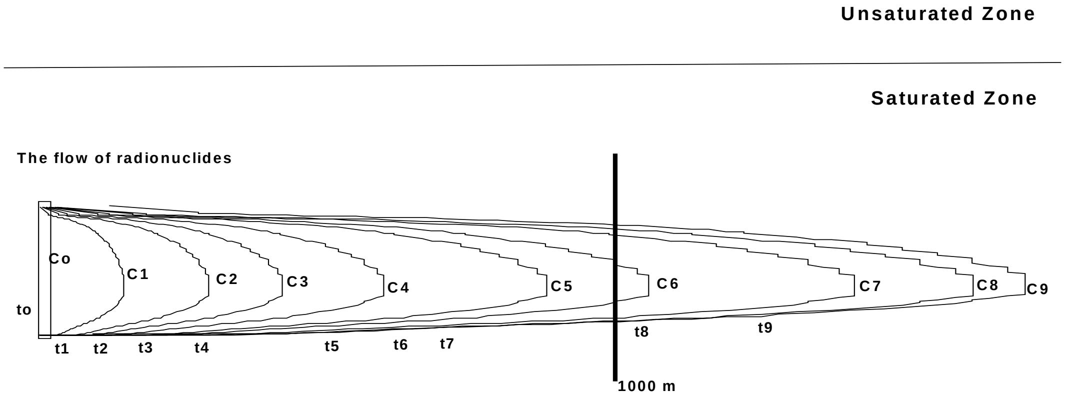

**FIGURE 6**. The concentration distribution of radionuclides.

Each wave is formed in a different period of time and has lower concentration than the previous wave and higher concentration than the next wave; i.e., $C_o > C_1 > C_2 > C_3$, etc. At a 1000-m distance and at each period of time, the concentration of the radionuclide is equal to the collective concentration of this radionuclide in the waves that present during that time. The concentration of each radionuclide at different times at 1000 m is calculated according to Eq. (3) and is represented in Fig. 7.

The concentration of each radionuclide behaves in an exponential manner. Therefore, despite the decreases of initial concentration of a radionuclide during its migration in the aquifer, the concentration at point in the aquifer increases gradually with the time until it attains a maximum value followed by gradual decrease with increase of time. The increase of concentration results from the consecutive passage of waves at any point and decreases when they move to an adjacent distance. The straight line on the top of the exponential curves in each subfigure expresses the standard concentration limit of radionuclide in drinking water.

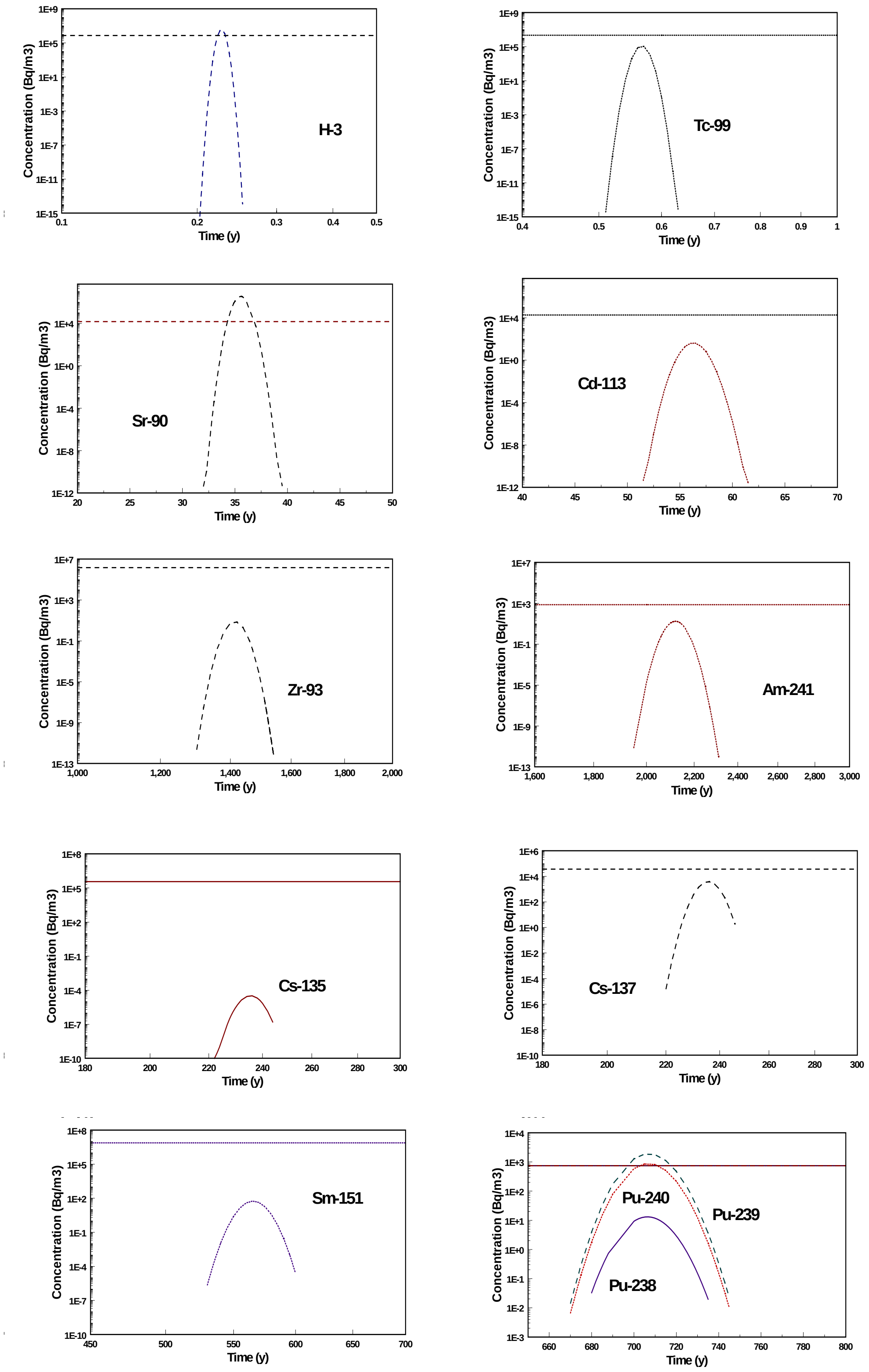

**FIGURE 7**. Maximum concentration of radionuclides at 1000 m

At the distance of concern, each radionuclide arrives depending on its retardation in soil to its maximum concentration at different times; the lower retardation, the faster arrival. This explains the faster appearance of H-3 followed by Tc-99 after 84 and 208 days, respectively (Table 7).

**TABLE 7**
**Maximum Concentration of Radionuclide with Corresponding Arrival Time**

| Radionuclide | Initial Concentration Entering the Saturated Zone ($Bq/m^3$) | Maximum Concentration ($Bq/m^3$) | Time of Arrival to Maximum Concentration (Year) |
|---|---|---|---|
| H-3 | 5.72E + 08 | 3.45E + 06 | 0.23 |
| Sr-90 | 2.16E + 11 | 3.55E + 06 | 36 |
| Zr-93 | 4.43E + 06 | 7.15E + 00 | 1420 |
| Tc-99 | 4.96E + 07 | 1.08E + 05 | 0.57 |
| Cd-113 | 2.93E + 07 | 3.91E + 01 | 56.5 |
| Cs-135 | 4.27E + 06 | 3.21E + 05 | 236 |
| Cs-137 | 2.35E + 11 | 3.86E + 03 | 236 |
| Sm-151 | 2.45E + 09 | 5.57E + 01 | 565 |
| Pu-238 | 5.83E + 08 | 1.30E + 00 | 705 |
| Pu-239 | 9.37E + 08 | 1.86E + 03 | 705 |
| Pu-240 | 4.53E + 08 | 8.51E + 02 | 705 |
| Am-241 | 1.04E + 09 | 1.79E + 01 | 2120 |

The value of maximum concentration of the radionuclide is influenced by initial concentration and retardation factor. Therefore, Sr-90 has highest maximum concentration equal 3.55E + 06 $Bq/m^3$ after 36 years. This is not the case of Cs-137 with highest initial concentration of 2.35E + 11 $Bq/m^3$ and high retardation, and arrives after 236 years with concentration of 3.86E + 03 $Bq/m^3$.

On the other hand, Pu-238, Pu-239, and Pu-240 have the arrival time of 565 years, but with different concentrations depending on their initial concentration of 1.30E + 00, 1.86E + 03, and 8.51E + 0,2 respectively.

The objective of this work was to evaluate the groundwater pollution resulting from the possibility of radioactive waste released from the settling tank. The maximum concentration of radionuclides in the aquifer at 1000 m was our concern. This evaluation was performed by comparing the maximum concentration of a radionuclide with its corresponding standard concentration limit in the drinking water. This evaluation is presented in Table 8.

H-3, Sr-90, Pu-239, and Pu-240 are the radionuclides that reach the 1000-m distance with a concentration value above the standard limit of drinking water.

H-3 radionuclide reaches the point of concern after 91 days. No prevention measures could be done to avoid this migration.

Sr-90, Pu-239, and Pu-240 reach the point of concern after 39 days for Sr-90 and 769 for any Pu isotopes. This duration of time allows the possible institution of remedial measures. Nevertheless, some published data provide evidence that “the rate of migration of some of the most dangerous constituents of the waste, such as plutonium and americium, is much faster than anticipated”[25]. This results can be explained by the formation of the colloid particulate $PuO_2$ which could be transported more rapidly than soluble species of plutonium[26].

**TABLE 8**
**Comparison of the Maximum Concentration of Radionuclides at 1000 m with the Standards Limit of Radionuclides in Water[2,3]**

| Radionuclide | Maximum Concentration ($Bq/m^3$) at 1000 m | Standard Limit in Drinking Water ($Bq/m^3$)[2] |
|---|---|---|
| H-3 | 3.45E + 06 | 3.70E + 07* |
| Sr-90 | 3.55E + 06 | 1.85E + 04 |
| Zr-93 | 7.59E + 00 | 1.48E + 06 |
| Tc-99 | 1.08E + 05 | 2.22E + 06 |
| Cd-113 | 3.91E + 01 | 1.48E + 04 |
| Cs-135 | 3.21E – 05 | 7.40E + 06 |
| Cs-137 | 3.86E + 03 | 3.70E + 05 |
| Sm-151 | 5.57E + 01 | 3.70E + 04 |
| Pu-238 | 1.30E + 00 | 7.40E + 02 |
| Pu-239 | 1.86E + 03 | 7.40E + 02 |
| Pu-240 | 8.51E + 02 | 7.40E + 02 |
| Am-241 | 1.79E + 01 | 7.40E + 02 |

***** This value refers to EPA (Safe Drinking Water Act)[3].

## Recommended First Steps for the Remedial Action

Simples processes can be recommended with low cost and with low efforts to remedy the possible pollution and keep it as low as possible. Initially, the underground tanks should be tested to know the real situation. If release is detected, repairing or replacing these tanks is required with the consideration of the standards regulations established[27]. Meanwhile, the piezometric wells that already exist around the reactor can be used as test wells with no additional cost. These piezometric wells are lies on the circle designed in Fig. 1. These data collected regularly are necessary to examine the effect of natural attenuation of different radionuclides, which helps to predict their migration time[28,29]. On the other hand, in the case of Sr-90 release, “phytoremediation techniques” may be used[30,31]. If the plume crossed the safe limit of the site — especially for Pu-239, Pu-240, and Am-241 — Subsurface Barrier or Reactive Barrier may be used[32]. If there is no availability for using these barriers, Pump and Treat techniques can be efficient for cleaning the aquifer to some safe extent[33].

These results study the migration processes of radionuclides that can leak from the settling tank and which have the half-life between 12 and 50,000 years. These radionuclides represent a real environmental threat. Therefore, this possibility should be studied carefully to establish all possible and available remediation measures to prevent any possible hazard.

## ACKNOWLEDGMENT

The authors would like to express special thanks to Prof. Dr. Anas El-Nagar (Prof. Emirates from the Egyptian Atomic Energy Authority) for reviewing this work and Prof. Hisham Aly, Ex-President of the Atomic Energy Authority for valuable remarks. Also, the authors would to thank Mr. Salah abd-El-Wahab (teacher assistant in the Atomic Energy Authority in Egypt) for his help in collecting water samples.
